# Lobectomy versus sublobar resection for stage I (T1‐T2aN0M0) small cell lung cancer: A SEER population‐based propensity score matching analysis

**DOI:** 10.1002/cam4.5568

**Published:** 2022-12-25

**Authors:** Ning Zhou, Lingqi Yang, Bo Zhang, Shuai Zhu, Huandong Huo, Jinling He, Lingling Zu, Zuoqing Song, Song Xu

**Affiliations:** ^1^ Department of Lung Cancer Surgery Lung Cancer Institute, Tianjin Medical University General Hospital Tianjin China; ^2^ Tianjin Key Laboratory of Lung Cancer Metastasis and Tumor Microenvironment Lung Cancer Institute, Tianjin Medical University General Hospital Tianjin China

**Keywords:** small cell lung cancer, stage I, surgery

## Abstract

**Objective:**

This study evaluated whether sublobar resection (sub‐L) is non‐inferior to lobectomy (L) for stage I (T1‐T2aN0M0) small cell lung cancer (SCLC) regarding long‐term overall survival (OS).

**Methods:**

Clinicopathological and prognostic data of patients with stage I (pT1‐T2aN0M0) SCLC were retrieved. Kaplan–Meier curves and Breslow tests were performed for the assessment of OS. Propensity score matching (PSM) analysis was used to mediate the inherent bias of retrospective researches.

**Results:**

A total of 188 patients with stage I SCLC were included in this study after PSM. For resected stage I SCLC, surgery plus adjuvant therapy was related to a better OS compared with surgery only (*p* = 0.016). For resected stage I SCLC, no matter adjuvant therapy was performed or not, no significant difference was observed in long‐term OS between the L and sub‐L groups (*p* = 0.181). Further subgroup analysis demonstrated that the OS disadvantage of sub‐L over L was not statistically significant for stage I SCLC patients underwent surgery only (*p* = 0.653), but also for the patients underwent surgery plus adjuvant therapy (*p* = 0.069). Moreover, in the subgroup analyses according to TNM stage (IA and IB), sex (male and female), and age (≥70 and <70 years), OS did not differ between the L and sub‐L groups except in female patients (*p* = 0.008). Multivariate Cox regression analysis indicated that adjuvant therapy was positively associated with OS.

**Conclusions:**

Surgery plus adjuvant therapy confers a better survival benefit than surgery only for stage I SCLC patients. However, as far as the range of surgical resection is concerned, sublobar resection may be non‐inferior to lobectomy regarding OS. Our study could conduce to the development of optimal therapeutic strategies for stage I SCLC patients. Further validation is warranted in larger retrospective and prospective cohort studies.

## Graphical abstract

This study evaluated the non‐inferior role of sublobar resection to lobectomy for stage I (T1‐2aN0M0) SCLC. We found that lobectomy was superior to sublobar resection in OS (A) and LCSS (C) before PSM, but no significant difference was observed between sublobar resection and lobectomy in OS (B) and LCSS (D) after PSM.

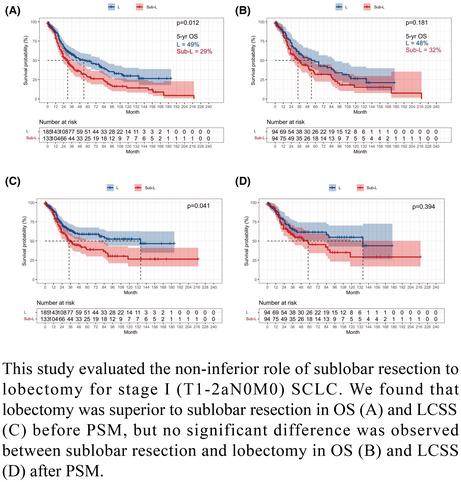

## 1 INTRODUCTION

Lung cancer is one of the commonest cancer types and leads to the largest number of cancer‐related death.^1^ Small cell lung cancer (SCLC), accounting for approximately 15% of all lung cancers, is the poorest prognosis lung cancer and is significantly correlated with smoking.^2^, ^3^ The highly aggressive growth pattern and early metastasis of SCLC result in a very poor prognosis for these patients.^2^, ^4^, ^5^

The therapeutic strategies for SCLC vary according to pathological stage. However, the vast majority of patients with SCLC are diagnosed with advanced stage at which surgery is not indicated. Surgery and radiation therapy are primarily recommended for the treatment of early‐stage (T1‐2N0M0) SCLC, which takes a proportion of 5% SCLC cases. Although surgery showed no benefits initially, its role in the treatment of SCLC has gradually improved in past three decades,^3^ especially for stage I disease.^6^ Several population‐based data analyses showed that the 5‐year survival rate of patients with stage I SCLC can reach 50% after complete pathological R0 resection.^7^ In addition, adjuvant chemotherapy is recommended after surgical resection.^8^

Previous studies suggest that sublobar resection achieves similar overall survival (OS) in stage I NSCLC than lobectomy.^9^, ^10^ Although surgery being the proven effective for the treatment of SCLC, the optimal surgical modality remains unclear. Schreiber et al. reported that, compared with sub‐lobar resection, lobectomy is positively related to OS in limited stage SCLC.^11^ Varlotto et al. found that lobectomy leads to superior survival in early‐stage SCLC.^12^ Susan et al. demonstrated that lobectomy leads to a superior OS than sublobar resection and pneumonectomy.^13^ However, inconsistencies in previous studies limit the reliability of this conclusion. Therefore, identifying the optimal surgical modality for stage I SCLC is important. This study evaluated the non‐inferior role of sublobar resection to lobectomy, for stage I (T1‐2aN0M0) SCLC regarding long‐term OS by using Surveillance, Epidemiology, and End Results (SEER) database and propensity score matching (PSM) analysis.

## 2 MATERIALS AND METHODS

### 2.1 Patient selection

Patients were selected from the SEER database of the National Cancer Institute. Patients were recruited according to the following criteria: (1) diagnosis of SCLC between 1998 and 2018 from the SEER database; (2) stage I SCLC with tumors ≤4 cm, which was corrected based on the AJCC (eighth version); (3) complete follow‐up information; (4) no lymph node metastasis (N0). Patients were ruled out based on the following criteria: (1) primary site was bilateral and in the main bronchus; (2) tumor size codes 990 or 996–999; and (3) tumor extent codes 950, 980, or 999. Finally, 318 patients were included in the study.

Clinicopathological and prognostic data was extracted from SEER database, including patients' age, gender and race; tumor's size, location, and stage; therapeutic details. Patients were classified into two subgroups, lobectomy group (L) and sub‐lobar resection group (sub‐L; wedge/segmentectomy). PSM was performed to impair the effect of inherent bias in the baseline characteristics of patients in the two subgroups.

### 2.2 Statistical analysis

Chi‐square test was performed to assess differences of clinicopathological characteristics between two subgroups. Kaplan–Meier curves and Breslow tests were utilized to compare the different OS and lung cancer specific survival (LCSS) between two subgroups. In addition, Cox regression analysis (univariate and multivariate) was used respectively to explore the independent prognostic factors with the cutoff *p* value of 0.05. In present research, the surgical allocation from SEER database did not allocate at random.

PSM was utilized to make the baseline characteristics of L and sub‐L groups consistent. During the PSM process, variables were identified from the acquirable clinicopathological characteristics related to OS: age, gender, race, tumor location, tumor size, chemotherapy, radiotherapy, and dissected regional lymph nodes. A 1:1 ratio of matched cohorts was created through pairing patients who treated with lobectomy and sub‐lobar resection, then the Breslow test was used to evaluate the OS difference between the L and sub‐L groups according to therapeutic strategy (surgery only and surgery plus adjuvant therapy), stage (stage IA or stage IB), age (≥70 years old, <70 years old), and gender (male, female).

All analyses were achieved by utilizing SPSS software (version 22.0), and the survival curve was generated using the “survminer” package in R software (version 4.1.2). All tests were two‐sided, and the *p* value <0.05 was defined statistically significant.

## 3 RESULTS

### 3.1 Patient clinicopathological characteristics

In total 318 patients with stage I SCLC were selected from SEER database (Figure S1). After PSM, 188 patients were chosen finally, and were divided into lobectomy group (*n* = 94) and sub‐lobar resection group (*n* = 94). The clinicopathological characteristics of patients before and after PSM are recorded in Table 1. No significant difference was observed between the two subgroups with the exception of tumor size, regional lymph node dissection, and radiotherapy (Table 1) before and after PSM. Among the patients who underwent lobectomy, 89.2% also underwent lymph node dissection, whereas the proportion was 45.9% in patients who received sub‐lobar resection.

**TABLE 1:** Clinicopathological characteristics of SCLCs before and after PSM

	No. (%) of patients before PSM			No. (%) of patients after PSM		
	Sub‐L (*N* = 133)	Lob (*N* = 185)	*p*	Sub‐L (*N* = 94)	Lob (*N* = 94)	*p*
Age (median)			0.422			0.769
≥70 years	65 (48.9)	82 (44.3)		43 (45.7)	41 (43.6)	
<70 years	68 (51.1)	103 (55.7)		51 (54.3)	53 (56.4)	
Gender			0.217			0.108
Male	69 (51.9)	83 (44.9)		51 (54.3)	39 (41.5)	
Female	64 (48.1)	102 (55.1)		43 (45.7)	55 (58.5)	
Race			0.901			0.592
White	116 (87.2)	159 (85.9)		81 (86.2)	76 (80.9)	
Black	10 (7.5)	14 (7.6)		8 (8.5)	12 (12.8)	
Other	7 (5.3)	12 (6.5)		5 (5.3)	6 (6.4)	
Stage			0.934			0.254
IA	90 (67.7)	126 (68.1)		64 (68.1)	72 (76.6)	
IB	43 (32.3)	59 (31.9)		30 (31.9)	22 (23.4)	
Location			0.569			0.492
Left upper	40 (30.1)	56 (30.3)		30 (31.9)	23 (24.5)	
Left lower	18 (13.5)	18 (9.7)		16 (17.0)	12 (12.8)	
Right upper	48 (36.1)	72 (38.9)		31 (33.0)	39 (41.5)	
Right middle	5 (3.7)	13 (7)		3 (3.2)	6 (6.4)	
Right lower	22 (16.6)	26 (14.1)		14 (14.9)	14 (14.9)	
Size			0.012			0.006
≤10 mm	15 (11.3)	14 (7.6)		6 (6.4)	14 (14.9)	
>10 mm, ≤20 mm	75 (56.4)	82 (44.3)		53 (56.4)	65 (69.1)	
>20 mm, ≤30 mm	37 (27.8)	64 (34.6)		29 (30.9)	13 (13.8)	
>30 mm, ≤40 mm	6 (4.5)	25 (13.5)		6 (6.4)	2 (2.1)	
Lymph nodes			<0.001			<0.001
Yes	61 (45.9)	165 (89.2)		57 (60.6)	74 (78.7)	
No	67 (50.4)	1 (0.5)		36 (38.3)	1 (1.1)	
Unknown	5 (3.7)	19 (10.3)		1 (1.1)	19 (20.2)	
Adjuvant therapy			0.811			0.526
Yes	89 (66.9)	121 (64.8)		68 (72.3)	63 (67.0)	
No/unknown	44 (33.1)	64 (35.2)		26 (27.7)	31 (33.0)	

Abbreviations: Lob, lobectomy; PSM, propensity score matching; Sub‐L, sub‐lobar resection.

### 3.2 Surgical outcome analysis

**FIGURE 1:**
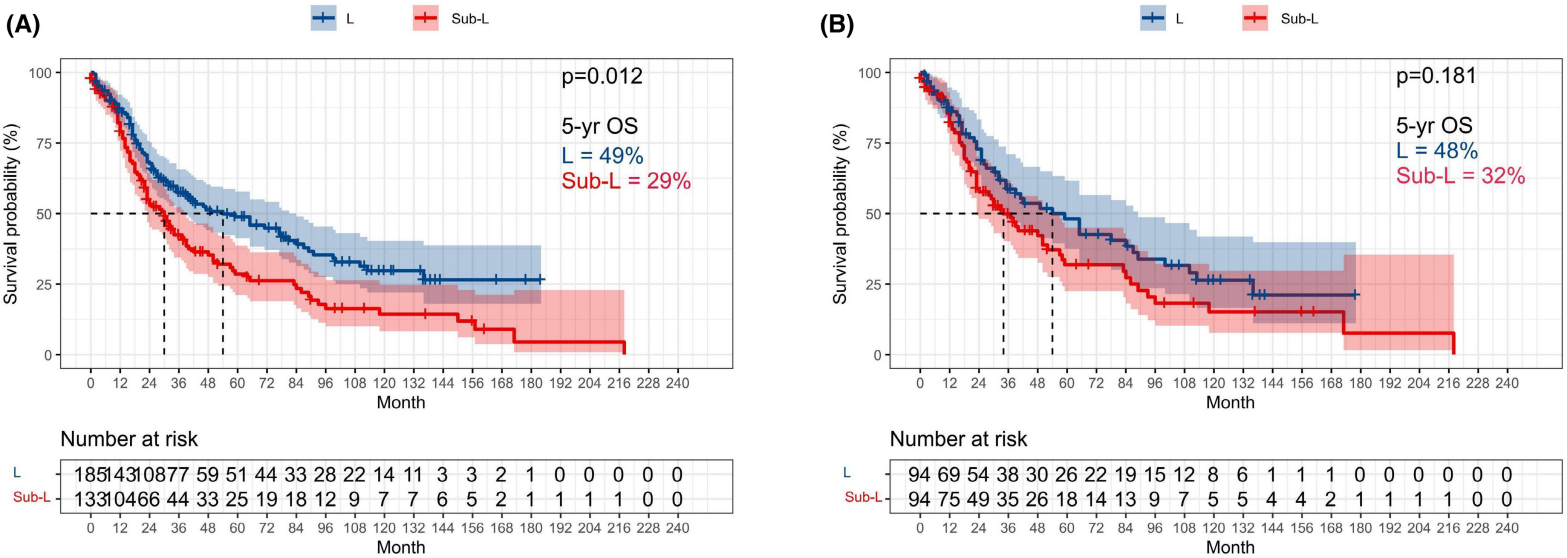
Kaplan–Meier survival curve of stage I SCLC patients. (A) Sublobar resection versus lobectomy in all patients before PSM; (B) Sublobar resection versus lobectomy in all patients after PSM.

We observed a significant difference on OS between the L and sub‐L groups before PSM (*p* = 0.012; 5‐year OS, L vs. sub‐L: 49% vs. 29%; Figure 1A). However, after PSM, no significant difference was found between the L and sub‐L groups (*p* = 0.181; 5‐year OS, L vs. sub‐L: 48% vs. 32%; Figure 1B). After PSM, the median survival time of L group was longer than sub‐L group (54 vs. 37 months). In addition, we also evaluated the role of surgery type on LCSS. We found that LCSS was significantly different between the L and sub‐L groups before PSM (*p* = 0.041), while no difference was observed after PSM (*p* = 0.394; Figure S1A,B).

### 3.3 Subgroup analysis according to adjuvant therapy

**FIGURE 2:**
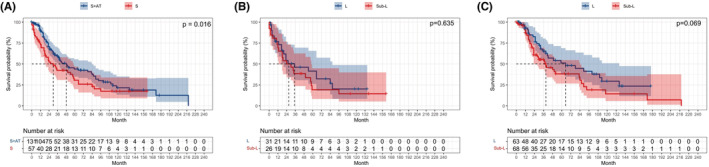
Kaplan–Meier survival curve of stage I SCLC patients stratified by therapy model. (A) Surgery plus adjuvant therapy versus surgery only; (B) Sublobar resection versus lobectomy in the patients with surgery alone; (C) Sublobar resection versus lobectomy in the patients with surgery plus adjuvant therapy. S + AT, Surgery plus adjuvant therapy; S, Surgery only.

We showed that sub‐lobar resection plays a non‐inferior role to lobectomy for all of the stage I SCLC. However, whether adjuvant therapy affects OS remains unclear. After PSM, surgery plus adjuvant therapy was associated with better survival than surgery only (*p* = 0.016; Figure 2A). Subgroup analysis was utilized to research the OS difference between patients who underwent surgery alone and others who underwent surgery plus adjuvant therapy. The results indicated that, for patients underwent surgery alone, lobectomy does not exhibit an obvious superiority over sub‐lobar resection (*p* = 0.653; Figure 2B). We then classified patients into two groups, lobectomy plus adjuvant therapy and sub‐lobar resection plus adjuvant therapy. It was also found that patients underwent lobectomy plus adjuvant therapy exhibited no prominent advantage in OS, compared with patients treated with sub‐lobar resection plus adjuvant therapy (*p* = 0.069; Figure 2C).

### 3.4 Subgroup analysis according to stage and tumor size

**FIGURE 3:**
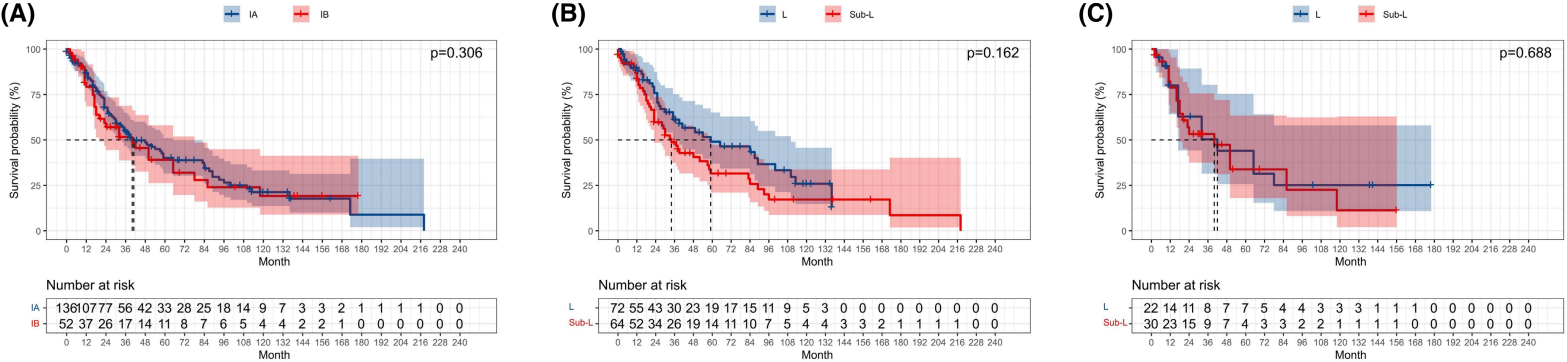
Kaplan–Meier survival curve of stage I SCLC patients stratified by stage. (A) OS of patients with stage IA versus stage IB; (B) Sublobar resection versus lobectomy in the patients with stage IA; (C) Sublobar resection versus lobectomy in the patients with stage IB.

**FIGURE 4:**
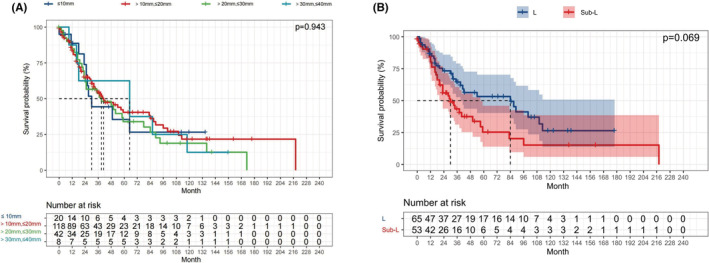
Kaplan–Meier survival curve of stage I SCLC patients stratified by tumor size. (A) OS of patients with *T* ≤ 10 mm, 10 < *T* ≤ 20 mm, 20 < *T* ≤ 30 mm, and 30 < *T* ≤ 40 mm; (B) Sublobar resection versus lobectomy in patients with 10 < *T* ≤ 20 mm.

Survival analysis according to stage was performed after classifying patients into stage IA and IB groups. No prominent difference was found between the IA and IB groups (*p* = 0.306; Figure 3A). We also observed the similar OS between the L and sub‐L groups among patients with stage IA (*p* = 0.162; Figure 3B) and those with stage IB (*p* = 0.688; Figure 3C). Analysis based on tumor size showed no obvious variation between patients with *T* ≤ 10 mm, 10 < *T* ≤ 20 mm, 20 < *T* ≤ 30 mm, and 30 < *T* ≤ 40 mm (*p* = 0.943; Figure 4A). In addition, there was also no significant variation in OS between the L and sub‐L groups in patients with *T* ≤ 10 mm (*p* = 0.187; Figure S2A), 10 < *T* ≤ 20 mm (*p* = 0.069; Figure 4B), 20 < *T* ≤ 30 mm, and 30 < *T* ≤ 40 mm (*p* = 0.131 and 0.737, respectively; Figure S2B,C).

### 3.5 Subgroup analysis according to age and gender

**FIGURE 5:**
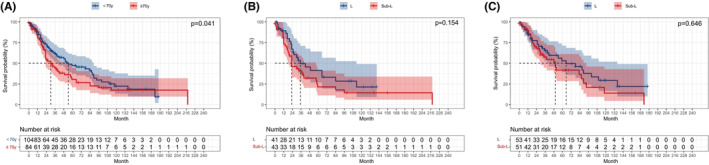
Kaplan–Meier survival curve of stage I SCLC patients stratified by age. (A) OS of patients with age ≥70 years versus age <70 years; (B) Sublobar resection versus lobectomy in patients with age ≥70 years; (C) Sublobar resection versus lobectomy in patients with age <70 years.

**FIGURE 6:**
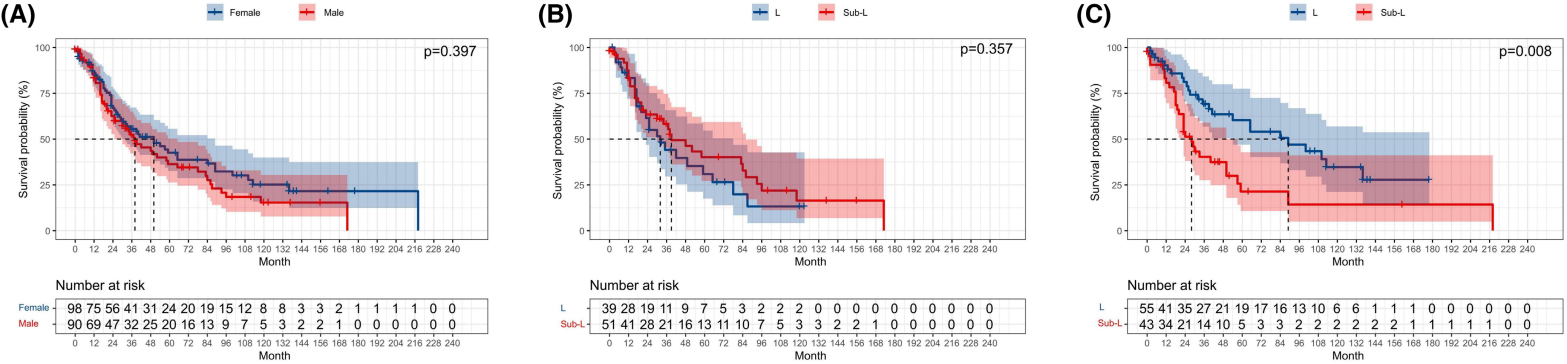
Kaplan–Meier survival curve of stage I SCLC patients stratified by gender. (A) OS of male patients versus female patients; (B) Sublobar resection versus lobectomy in the male patients; (C) Sublobar resection versus lobectomy in the female patients.

For survival analyses according to age, patients were classified into ≥70 years and <70 years. Elder patients showed a poorer prognosis than younger patients (*p* = 0.041; Figure 5A). In patients ≥70 years, we observed the similar OS between the L and sub‐L groups (*p* = 0.154; Figure 5B). The same result was also found in patients <70 years of age (*p* = 0.646; Figure 5C). Analysis according to gender showed no obvious variation between men and women (*p* = 0.397; Figure 6A). In men, the equivalent OS was observed between the L and sub‐L groups (*p* = 0.357; Figure 6B). However, among women, a prominently superior OS was observed in the L group than in the sub‐L group (*p* = 0.008; Figure 6C).

### 3.6 Cox regression analysis

**FIGURE 7:**
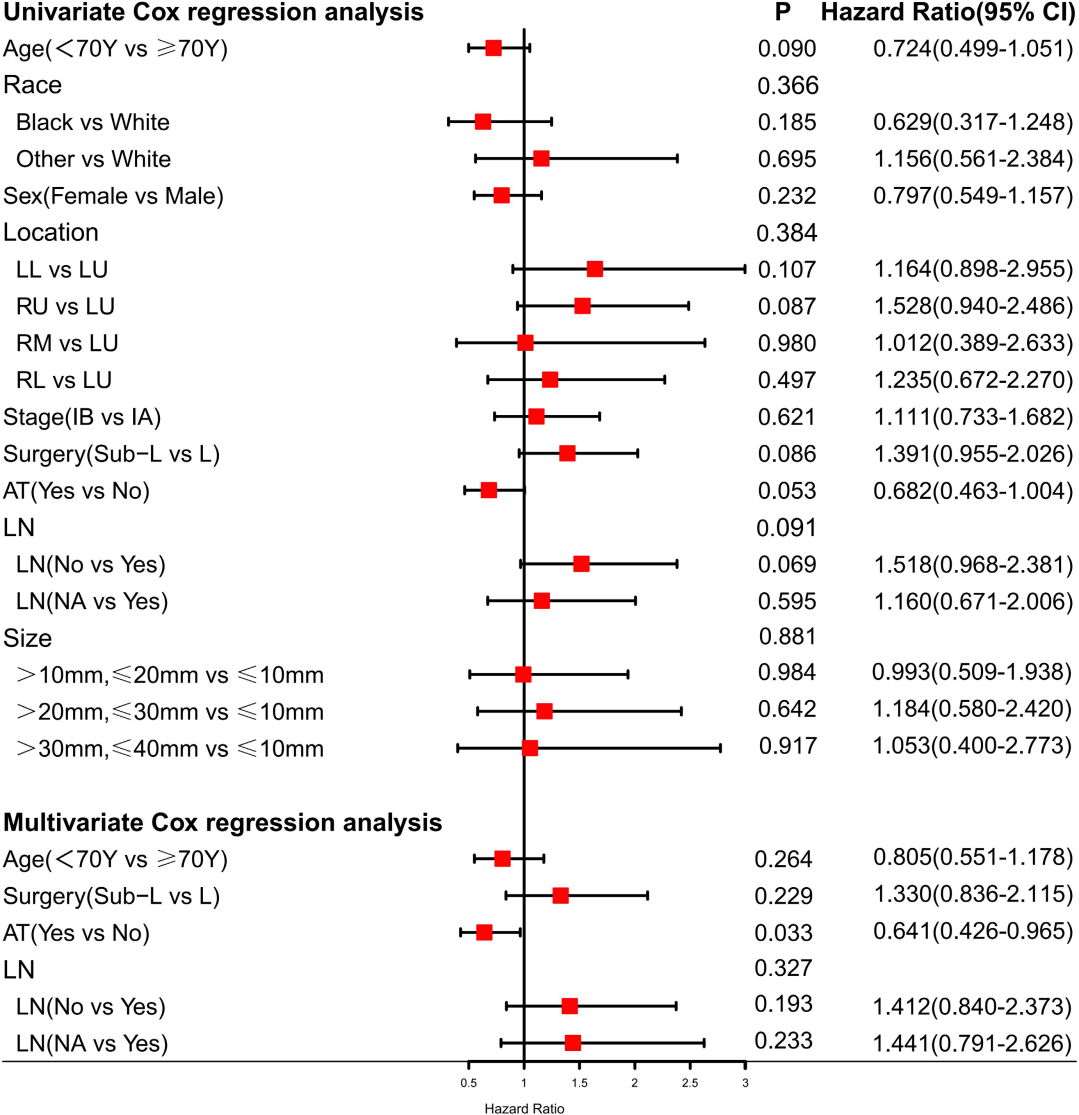
Univariate and multivariate Cox regression analysis of factors affecting overall survival.

Univariate and multivariate Cox regression analysis was utilized respectively to research the underlying risk factors that were associated with OS in stage I SCLC patients (Figure 7). Multivariate Cox regression analysis indicated that adjuvant therapy was positively related to a better OS (*p* = 0.033), whereas other factors, including age, gender, race, sites, tumor size, and surgery type, were not significantly related to OS (Figure 7). In the Univariate Cox regression analysis, none of those factors were associated with OS (Figure 7).

## 4 DISCUSSION

SCLC is one of the most fatal tumors and accounts for 15% of all lung cancers.^2^ Although the incidence of SCLC has gradually decreased in advanced countries for the past few years, its morbidity in other countries is gradually increasing.^14^ In the past decades, surgical treatment is not recommended for patients with SCLC. Therapeutic efficacy of surgery for patients with early‐stage SCLC was confirmed in studies showing that surgical treatment leads to prolonged OS in limited stage SCLC.^15^, ^16^, ^17^, ^18^, ^19^, ^20^ Although lobectomy confers a survival advantage compared with sub‐lobar resection, evidence is lacking in stage I SCLC. Because of the low proportion of stage I SCLC patients, we used the SEER database to study the efficacy of surgical treatment for patients with stage I SCLC by comparing the OS of patients undergoing sub‐lobar resection and lobectomy using PSM analysis.

The present study enrolled 318 stage I SCLC patients. It was found that no significant different OS was observed between the L and sub‐L groups including all stage I SCLC patients after the PSM analysis. Studies indicate that survival is worse after sub‐lobar resection than after lobectomy in SCLC patients.^11^, ^12^, ^13^, ^21^, ^22^, ^23^ However, previous studies included patients with other stages in addition to stage I SCLC or did not perform PSM, which limits the significance of the conclusion. The present data suggest that survival was significantly better in stage I SCLC patients treated with lobectomy than in those treated with sub‐lobar resection before PSM, which is coherent with previous researches. However, the advantage of lobectomy decreased after PSM, which indicated a non‐inferior survival benefit of sublobar resection over lobectomy for resected stage I SCLC patients. Subgroup analysis suggested that two treatment modalities exhibit no significantly different therapeutic efficacy, no matter patients underwent surgery only or surgery plus adjuvant therapy. In addition, a previous study suggested that segmentectomy was non‐inferior to lobectomy, and both of lobectomy and segmentectomy played a superior role to wedge resection in limited‐stage SCLC.^24^ In present study, we also found similar conclusion in stage I patients before PSM (Figure S3A), while the superior OS was observed only in segmentectomy group compared with wedge resection group after PSM (Figure S3B). In addition, the LCSS of patients who underwent lobectomy, segmentectomy, or wedge resection was also calculated. Before PSM, wedge resection was inferior to lobectomy and segmentectomy (*p* = 0.008 and 0.012 respectively; Figure S4A). Wedge resection played a non‐inferior role to lobectomy after PSM (*p* = 0.184), but was still inferior to segmentectomy (*p* = 0.048; Figure S4B). Of note, segmentectomy played a non‐inferior role to lobectomy before and after PSM (*p* = 0.128 and 0.186 respectively; Figure S4A,B).

Chemotherapy and radiotherapy have been proven effective for the treatment of limited stage SCLC,^25^ and combining chemotherapy and radiotherapy contributes to a better survival benefit.^26^, ^27^, ^28^ A research of Pignon et al. indicated that chemotherapy alone is related to a worse survival than chemoradiation regarding the 3‐year OS.^29^ Another meta‐analysis reported that, compared with chemotherapy alone, chemoradiation provides a better clinical benefit regarding the 2‐year intrathoracic tumor control rate.^30^ In this study, a significant better OS was observed for the surgical patients who treated with adjuvant therapy than those who underwent no adjuvant therapy. Our result is coherent with the previous researches which suggest that adjuvant therapy was positively related to a prolonged OS in stage I SCLC patients. Multivariate Cox regression analysis also identified adjuvant therapy as a prominent independent factor related to OS.

Our study also had several limitations. First, despite the use of PSM, inherent bias is inevitable due to the inscape of retrospective study. Second, the SEER database lacks some information, such as smoking status, resection margins (R0, R1, or R2), tumor site (central or peripheral), progression‐free survival (PFS) as well as treatments after disease relapse/ metastasis, which play important roles in survival. Third, the number of stage I SCLC patients in the SEER database is limited, and this was further decreased after PSM. Fourth, some of the clinical information is incomplete, including the number of regional nodes and the information on adjuvant/neoadjuvant therapy. In addition, the role of radiotherapy could not be assessed precisely because it is always accompanied with chemotherapy. Lastly, owing to the insufficient number of patients treated with sub‐lobar resection, a comparison of segmentectomy with wedge resection after PSM analysis was not possible.

Altogether, sublobar resection may play a non‐inferior role to lobectomy regarding OS in stage I SCLC patients who received post‐operative adjuvant therapy. This study may contribute to the development of optimal surgical therapeutic strategies for stage I SCLC patients. Further validation is warranted in larger retrospective and prospective cohort studies.

## AUTHOR CONTRIBUTIONS

**Ning Zhou:** Conceptualization (lead); data curation (lead). **Lingqi Yang:** Conceptualization (equal). **Bo Zhang:** Writing – original draft (equal). **Shuai Zhu:** Visualization (equal). **Huandong Huo:** Visualization (equal). **Jinling He:** Visualization (equal). **Lingling Zu:** Visualization (equal). **Zuoqing Song:** Supervision (equal). **Song Xu:** Supervision (lead).

## FUNDING INFORMATION

The present study was funded by the National Natural Science Foundation of China (82172776), Tianjin Science and Technology Plan Project (19ZXDBSY00060) and (303078100412), Tianjin Key Medical Discipline (Specialty) Construction Project (TJYXZDXK‐061B), and Diversified Input Project of Tianjin National Natural Science Foundation (21JCYBJC01770).

## CONFLICT OF INTEREST

The authors declare that no conflict of interest exists in this study.

## ETHICS STATEMENT

The present research was performed by utilizing SEER database which is a public database for all the researchers. And this study did not involve any human tissue samples. Consequently, our study acquires an exemption from the ethical board.

## Supporting information


Figure S1

Figure S2

Figure S3

Figure S4

## Data Availability

Data sharing is not applicable to this article as no new data were created or analyzed in this study.
