# Supplementary figures and images for: Lobectomy versus sublobar resection for stage I (T1‐T2aN0M0) small cell lung cancer: A SEER population‐based propensity score matching analysis
Source: Cancer Med. 2022 Dec 25;12(7):7923–31. doi: 10.1002/cam4.5568 (PMC10134369; doi:10.1002/cam4.5568)

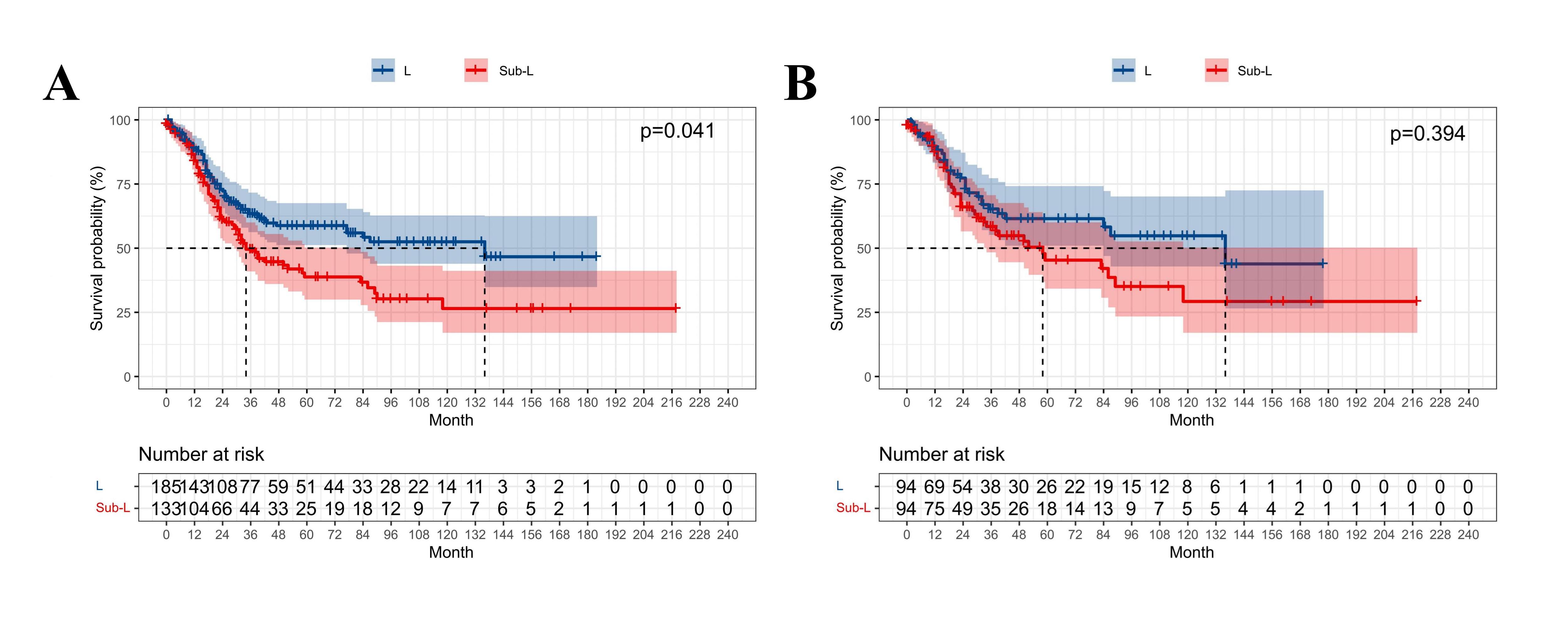

Supplement: Supplementary file 1 — Figure S1 [file CAM4-12-7923-s004.jpg]

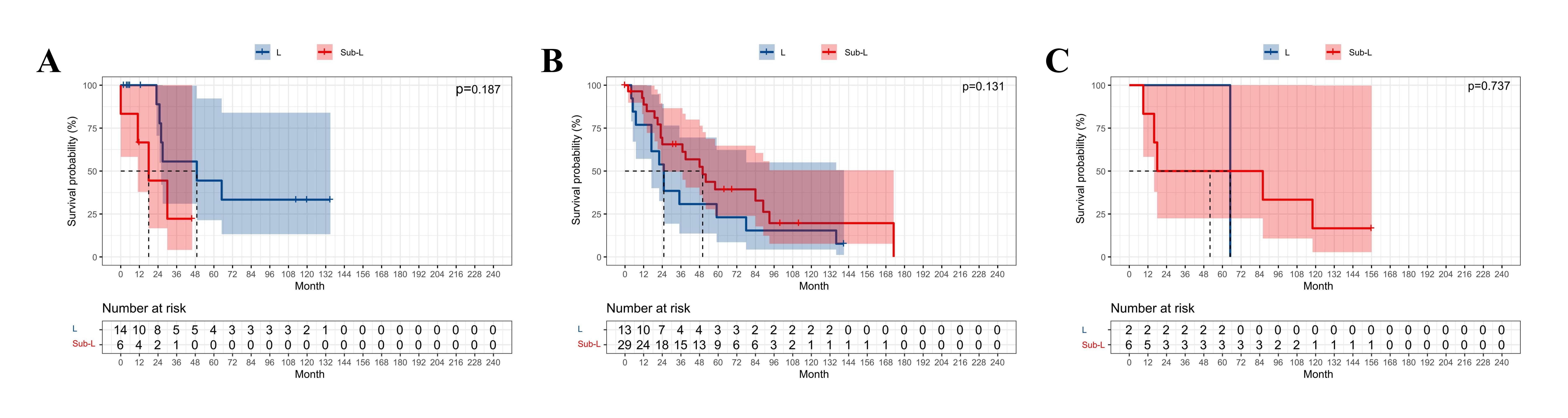

Supplement: Supplementary file 2 — Figure S2 [file CAM4-12-7923-s002.jpg]

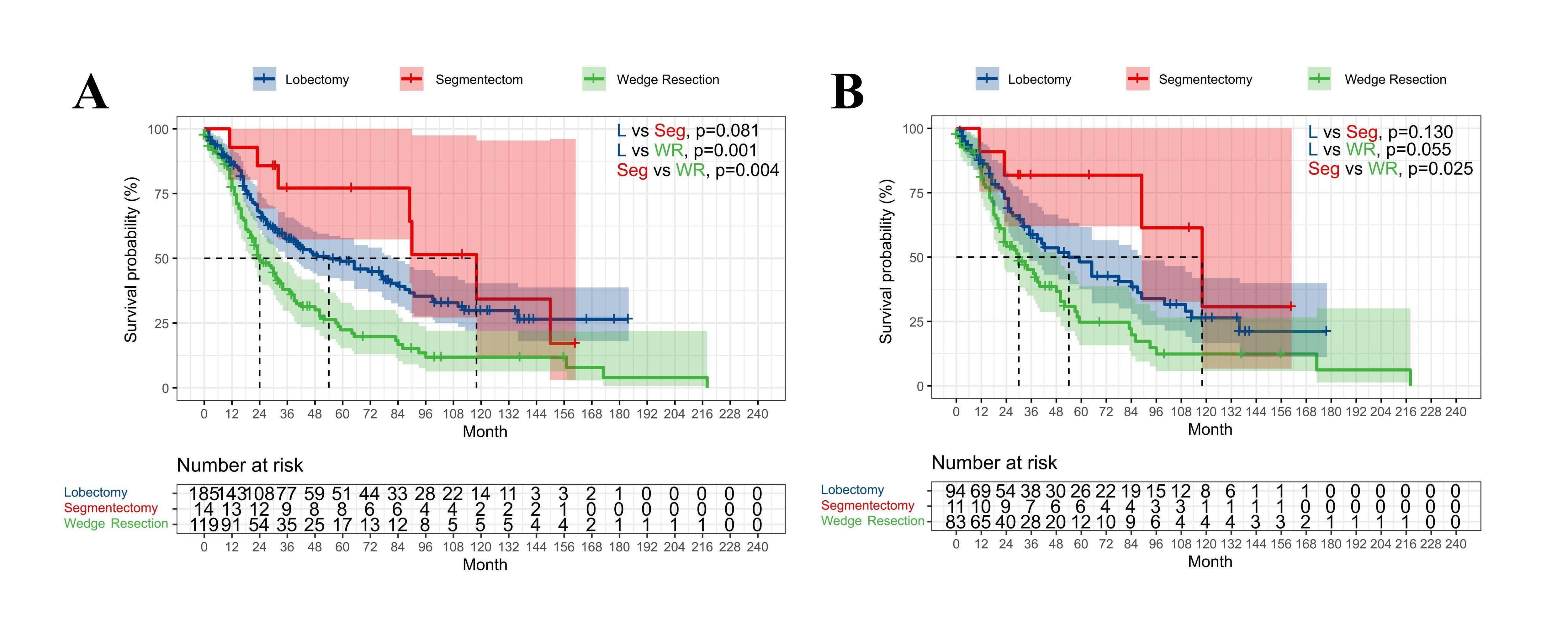

Supplement: Supplementary file 3 — Figure S3 [file CAM4-12-7923-s003.jpg]

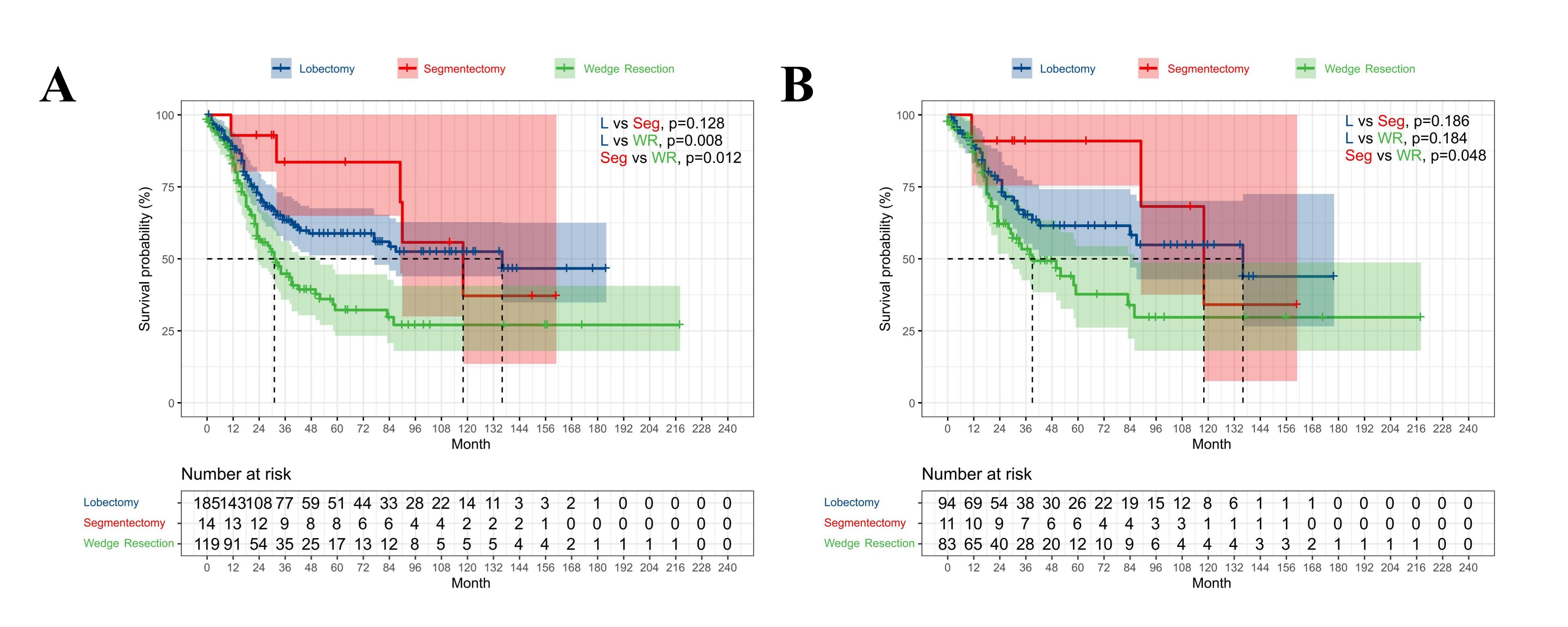

Supplement: Supplementary file 4 — Figure S4 [file CAM4-12-7923-s001.jpg]
